# A Spontaneous Dominant-Negative Mutation within a 35S::*AtMYB90* Transgene Inhibits Flower Pigment Production in Tobacco

**DOI:** 10.1371/journal.pone.0009917

**Published:** 2010-03-29

**Authors:** Jeff Velten, Cahid Cakir, Christopher I. Cazzonelli

**Affiliations:** 1 Plant Stress and Water Conservation Laboratory, United States Department of Agriculture - Agricultural Research Service, Lubbock, Texas, United States of America; 2 Australian Research Council - Centre of Excellence in Plant Energy Biology, Research School of Biology, Australian National University, Canberra, Australian Capital Territory, Australia; University of Melbourne, Australia

## Abstract

**Background:**

In part due to the ease of visual detection of phenotypic changes, anthocyanin pigment production has long been the target of genetic and molecular research in plants. Specific members of the large family of plant myb transcription factors have been found to play critical roles in regulating expression of anthocyanin biosynthetic genes and these genes continue to serve as important tools in dissecting the molecular mechanisms of plant gene regulation.

**Findings:**

A spontaneous mutation within the coding region of an Arabidopsis 35S::*AtMYB90* transgene converted the activator of plant-wide anthocyanin production to a dominant-negative allele (PG-1) that inhibits normal pigment production within tobacco petals. Sequence analysis identified a single base change that created a premature nonsense codon, truncating the encoded myb protein. The resulting mutant protein lacks 78 amino acids from the wild type C-terminus and was confirmed as the source of the white-flower phenotype. A putative tobacco homolog of *AtMYB90* (*NtAN2*) was isolated and found to be expressed in flower petals but not leaves of all tobacco plants tested. Using transgenic tobacco constitutively expressing the *NtAN2* gene confirmed the NtAN2 protein as the likely target of PG-1-based inhibition of tobacco pigment production.

**Conclusions:**

Messenger RNA and anthocyanin analysis of PG-1Sh transgenic lines (and PG-1Sh x purple 35S::*NtAN2* seedlings) support a model in which the mutant myb transgene product acts as a competitive inhibitor of the native tobacco NtAN2 protein. This finding is important to researchers in the field of plant transcription factor analysis, representing a potential outcome for experiments analyzing *in vivo* protein function in test transgenic systems that over-express or mutate plant transcription factors.

## Introduction

**Figure 1:**
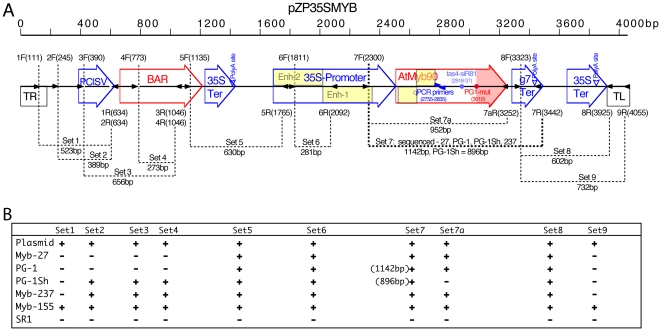
PCR scan across the T-DNA construct introduced into Myb-27. A. Map of the T-DNA containing a 35S::*AtMYB90* transgene introduced into *N. tabacum* to create the Myb-27 purple plant line: ‘TR’, right T-DNA border; ‘PClSV-Pro’, PClSV promoter; ‘BAR-Coding’, basta resistance gene; ‘35S-Ter’, CaMV 35S termination signal; ‘2xEnh35S-Pro’, CaMV 35S promoter with duplicated enhancer region; ‘*AtMYB90*-Coding’, Arabidopsis MYB90 gene; ‘g7-Ter’, termination signal from gene-7 of octopine T-DNA; ‘TL’, left T-DNA border. The small black arrows show PCR primers (primer identifiers listed above [forward] and below [reverse] each arrow) used to confirm the structure of the 35S::*AtMYB90* transgene in plant samples. Primer sets used are indicated by dashed lines (PCR product size, bp, in parenthesies). Set 7 indicates the area of the *Myb-27* and *PG-1 alleles* that was PCR amplified from transgenic plants and sequenced, with the red spot in *AtMYB90*-coding showing the location of the PG-1 nonsense (AAT->TAG, K172*) mutation (shaded area of the *AtMYB90* coding region indicates the amino acids missing from PG-1 and the DNA segment deleted in PG-1Sh). B. PCR results are alligned with the corresponding primer sets indicated in part A (numbered 1–9), with ‘+’ indicating a positive PCR band of the predicted size, and ‘-’ signifying no PCR product. The plasmid DNA used as a positive control, pZP35SMYB, is the binary construct used to generate the Myb-27 transgenic plant line. The remaining templates (total plant leaf DNA) are from the purple Myb-27 line, the white-flower PG-1 line, the white-flower PG-1Sh line and two additional independently derived purple transgenic tobacco lines (Myb-155 and Myb-237).

**Figure 2:**
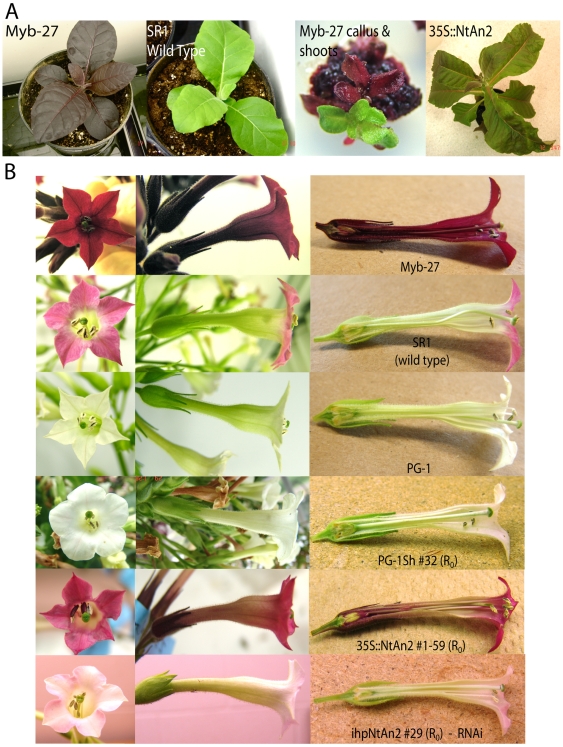
Photos displaying the phenotypes of transgenic plant lines used in this study. A. The Myb-27 transgenic plant line, wild type *N. tabaccum* cv SR1, Myb-27 callus with induced green and purple shoots and the NtAN2-1-59 line (35S::*NtAN2*). B. Flowers from the purple Myb-27 line, wild type *N. tabaccum* cv SR1, the dominant-negative white flower mutant PG-1 line, the shortened *Myb-27*, PG-1Sh (ransgenic line 32), the NtAN2 hairpin RNA (transgenic line 29) and the NtAN2-1-59 line. Flowers on the right were hand sectioned longitudinally to show internal components.

Anthocyanins represent a broad family of plant pigments that contribute to flower and fruit pigmentation [1], plant stress response [2], [3] and have been implicated as helpful nutrients that contribute to improved human health [4]. The production of anthocyanins and related pigments in plants has been the target of extensive genetic and molecular research and represents one of the better understood plant gene regulatory systems. Specific members of the Myb family of plant transcription factors have been found to play critical roles in controlling the expression of genes associated with anthocyanin production, often in conjunction with members of the basic helix-loop-helix (bHLH) and WD40 families of trans factors (e.g. [5], [6], [7], [8], [9], [10], [11], [12], [13]). A classic example of this form of gene regulation was originally identified through genetic mapping of maize mutations affecting seed-coat color. Many of these maize mutant alleles mapped to the C1 (MYB) [14], [15], R (bHLH) [16], [17], or PAC1 (WD40) [18] loci [19]. More recently, other examples of plant MYB genes in the R2R3 family [20], [21] have been found to play significant roles in controlling pigment production in flowers, fruit and vegetative tissues of several plant species [9], [22]. Transgenic ectopic over-expression of several of these MYB genes has been shown to dramatically impact anthocyanin accumulation, in many cases affecting pigmentation within plant species other than those from which the MYB transgenes originated [13], [23], [24], [25], [26], [27], [28], [29], [30], [31], [32], [33], [34]. Ectopic expression of either of two closely related Arabidopsis MYB genes, *AtMYB75* (*PAP1*) and *AtMYB90* (*PAP2*) in *Nicotiana tabacum* produced striking levels of anthocyanin pigmentation in most parts of transgenic plants, providing a clear visual indicator of transgene activity [35]. A similar dark purple 35S::*AtMYB90* transgenic tobacco line was created in this laboratory (Myb-27, Fig. 1 & 2) and used as test material in a visual screen for molecular mechanisms that can alter transgene expression levels and/or patterns during *in vitro* de-differentiated growth, and subsequent *de novo* shoot production, processes that are normally part of plant genetic transformation protocols. A single plant line (PG-1) regenerated from purple Myb-27 callus, was initially identified by a complete loss of the darkly pigmented phenotype of the parental line. Upon reaching maturity, the PG-1 line was found to display a white flower phenotype that differed from the dark purple flowers of MYB-27 and the lightly pigmented red flowers of wild-type tobacco [*N. tabacum*, cv SR1 [36]]. Genetic and molecular analysis of the PG-1 line indicate that both the loss of hyper-pigmentation and the white flower phenotype are the result of a spontaneous dominant-negative nonsense mutation within the coding region of the *AtMYB90* transgene. The observed dominant-negative white flower phenotype seen with the *PG-1* allele is similar to that reported in transgenic tobacco lines expressing the maize *C1-I* mutant allele [37]; and a wild type strawberry myb (*FaMYB1* [38]). The structure and properties of the *PG-1* dominant-negative mutation demonstrate a mechanism for manipulating Myb gene structure that can provide useful insight into the mechanisms by which MYB transcription factors function to regulate gene expression in plants.

## Results

### Myb-27: production and properties of the 35S::*AtMYB90* transgenic lines; callus propagation; and *de novo* shoot induction

The *AtMYB90* coding region, under control of a CaMV 35S promoter [39] and the T-DNA gene-7 transcription termination/polyadenylation signal sequence ([40], Fig. 1A), was introduced into tobacco (*N. tabacum* cv SR1) and resulting transgenic shoots screened visually for ectopic anthocyanin production. The Myb-27 line was selected as a purple shoot from callus associated with the initial Agrobacterium-treated tobacco leaf explants. Subsequent phosphinothricin treatment of R1 Myb-27 seedlings indicated that the line was not herbicide resistant, consistent with PCR scans spanning the introduced T-DNA (Fig. 1B). Other transgenic lines also chosen for their purple phenotypes (e.g. Myb-237 and Myb-155) were found to harbor functional glufosinate resistance genes (Fig. 1B). The transgenic line, Myb-27, was selected for additional analysis based upon its dominant, heavily pigmented phenotype (Fig. 2A). Although the purple Myb-27 plants grow more slowly than their wild-type tobacco parent under low light conditions (∼60 uMol quanta m^−2^ s^−1^), they otherwise display no obvious developmental or morphological changes. Actively growing cultured callus derived from surface sterilized hemizygous Myb-27 leaf material was found to display extensive anthocyanin pigmentation and was capable of producing new shoots, most of which displayed anthocyanin pigment patterns and levels similar to the parent Myb-27 plant (Fig. 2A).

### Myb-27 plants regenerated from callus can revert to a wild-type, green, phenotype

Of ∼100 plantlets regenerated and rooted from hemizygous purple Myb-27 callus, 4 completely lacked ectopic purple pigmentation (Fig. 2A). These 4 green regenerants were subsequently screened by PCR for the presence of the 35S::*AtMYB90* transgene (primer set 7a, Fig. 1A). Only one plant, designated line PG-1, gave a positive PCR signal, with the other three green plants apparently having lost the transgene during callus growth and/or plant regeneration. After reaching maturity the PG-1 line was found to display a white flower phenotype, producing flower petals that not only lacked the dark pigmentation of Myb-27 flowers, but also failed to produce the normal lightly pigmented red petals seen in wild-type tobacco (Fig. 2B).

### The *PG-1* locus contains a single-base, dominant-negative, nonsense mutation within the *AtMYTB90* transgene

Plants grown from seed of the selfed R_0_ PG-1 plant displayed an approximately 3∶1 ratio of white to pink flowered plants (29 white, 11 pink), results consistent with the original PG-1 transgenic plant being hemizygous for a single, dominant-negative, white-flower locus. The dominant-negative character of the *PG-1* allele was confirmed by crossing the PG-1 R_0_ plant to wild-type tobacco, producing an approximate 1∶1 ratio of white (18) to red (21) flower phenotypes in the resulting seedlings.

**Figure 3:**
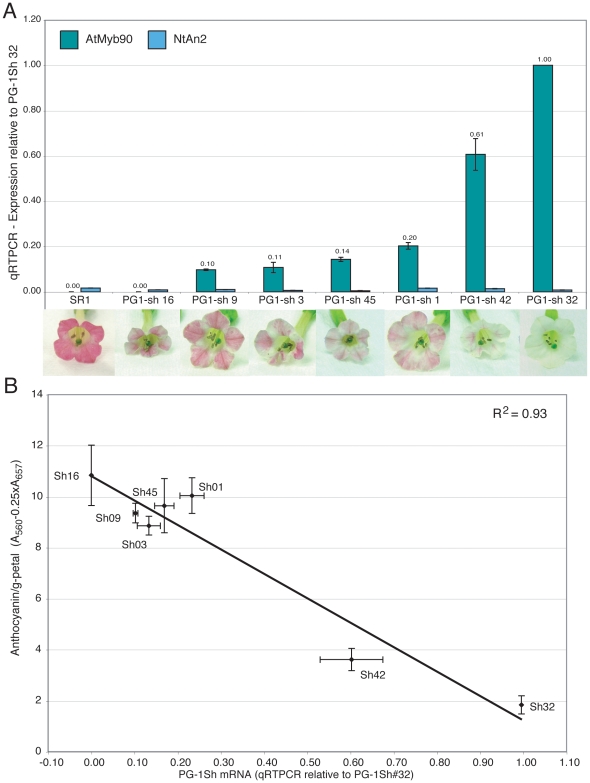
Analysis of anthocyanin levels and *AtMYB(90)* expression in PG-1Sh transgenic lines. A. Flower total RNA was used for qRTPCR determination of mRNA levels from the PG-1Sh transgene (purple) and the endogenous tobacco homolog, *NtAN2* (blue). All values (shown above the PG-1Sh bars) are reported relative to the mRNA level for the PG-1Sh transgene in line #32 and are the mean of 3 to 4 biological reps. The PG-1Sh transgene appears to be inactive in line #16. Photos of representative flowers from each plant line are shown below the graph. B. Spectrophotometically determined anthocyanin levels in flowers (n = 3 to 4) from the same transgenic lines were plotted against the relative PG-1Sh mRNA amounts shown in part A. PG-1Sh mRNA levels show an inverse correlation with anthocyanin content (R^2^ = 0.94), while an identical plot of anthocyanin content against *NtAN2* mRNA levels using the same flower RNA samples showed no correlation with pigmentation (R^2^ = 0.02).

**Figure 4:**
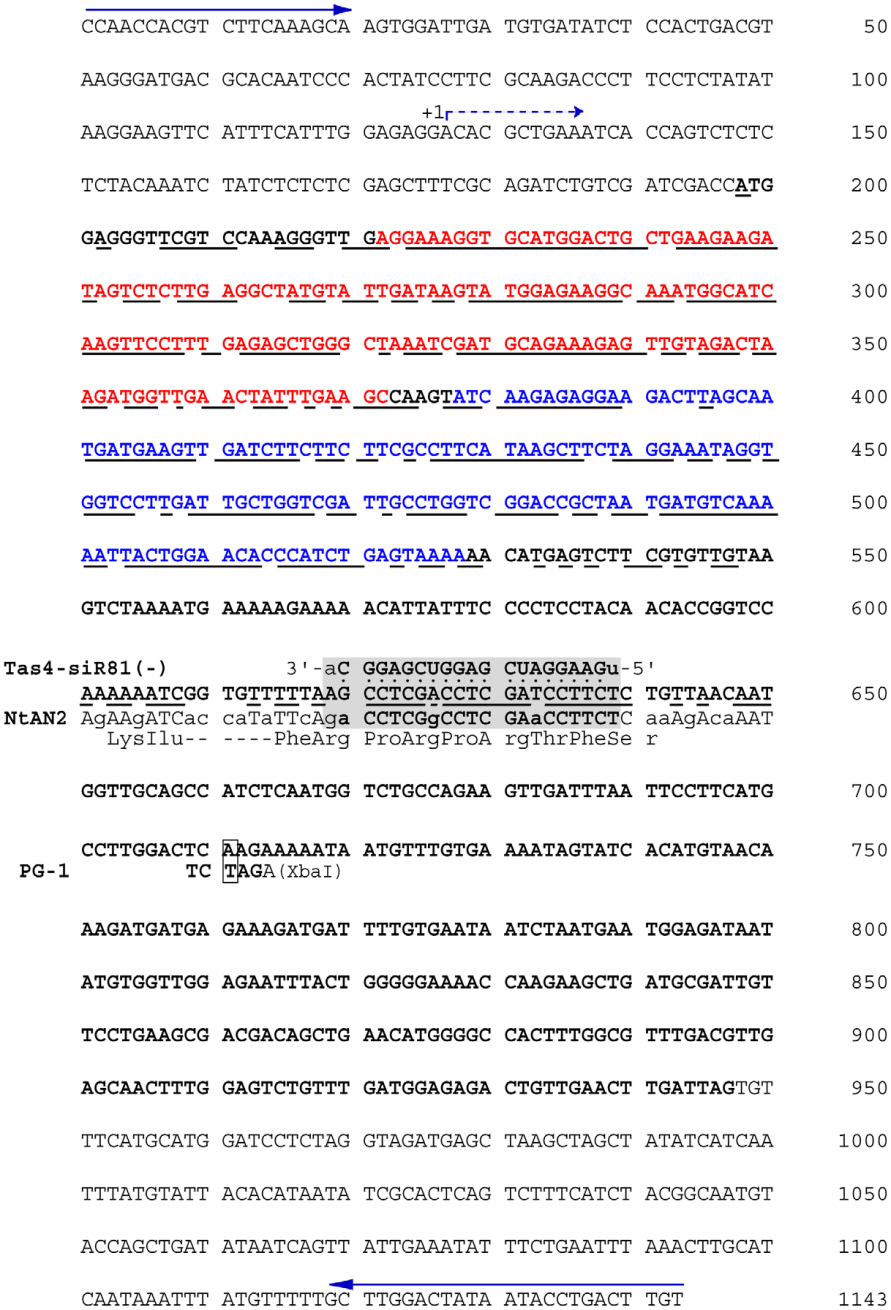
DNA sequence of the *AtMYB90* region within PG-1 and Myb-27 transgenic plants. The *AtMYB90* coding region is indicated by bold text (Red is Repeat 2, and Blue, Repeat 3) and the predicted transcription start site by a dashed arrow. PCR primers used to amplify the sequenced segment from total plant DNA are indicated by arrows. The PG-1 mutated codon is boxed (A to T mutation produces a new XbaI cut site). A TAS4-siR81(-) tasiRNA recognition site [43] is indicated (grey box). In the area of the recognition site the coresponding *NtAN2* DNA and predicted amino acid sequences are shown below (divergent bases, lower case). Areas of significant DNA homology between the *AtMYB90* and *NtAN2* sequences are underlined.

PCR analysis using primers targeting additional sites within the T-DNA used to create the Myb-27, and subsequent PG-1, transgenic lines failed to indicate any gross rearrangements of the PG-1 T-DNA relative to that present in Myb-27 plants (Fig. 1B). DNA isolated from Myb-27, PG-1 and Myb-237 lines was used to produce PCR products covering the area flanked by primer set 7 (extending from the 35S promoter to the g7 termination signal, Fig. 1A). Sequence derived from these PCR products indicated that, relative to the wild-type Myb-27 *AtMYB90* allele, the *PG-1* allele contains a single base change within the myb coding region. This mutation, an A to T transversion, converts an AAG (lysine) codon to a TAG (ocher) nonsense triplet at the 172^nd^ codon (Fig. 3), and is predicted to produce a truncated AtMYB90 protein that lacks the C-terminal 78 amino acids of the 249 amino acid AtMYB90 protein (Fig. 4). The A to T mutation also creates a new XbaI cleavage site (Fig. 4), allowing direct detection of the *PG-1* allele by XbaI digestion of PCR products from flanking primers, followed by electrophoretic separation of the resulting two DNA fragments. The new XbaI site was used to confirm the presence of the *PG-1* allele in all experiments involving PG-1 plant lines.

### The predicted PG-1 protein can produce a white-flower phenotype in tobacco

To test the hypothesis that the predicted shortened PG-1 protein is responsible for the observed white-flower phenotype, a new 35S::*AtMYB90* variant (PG-1 Short, or *PG-1Sh*) was generated and introduced into tobacco plants. The PG-1Sh construct lacks DNA encoding the 78 C-terminal amino acids downstream from the site of the *PG-1* mutant stop codon (Fig. 1A), and should produce the same shortened AtMYB90 protein as is predicted for the *PG-1* mutant allele. Transgenic tobacco lines expressing the PG-1Sh transgene displayed a range of flower color phenotypes, including plants with completely white flowers similar to those seen with the PG-1 line (Fig. 2B). Quantitative reverse-transcriptase PCR (qRTPCR) using mRNA from flowers of PG-1Sh lines chosen for their broad range in flower pigmentation indicated that expression of the PG-1Sh transgene was inversely proportional (R^2^ = 0.93) to flower anthocyanin pigment levels (Fig. 3A&B). These results support a model in which the *PG-1* or *PG-1Sh* gene product interferes competitively with the normal functioning of an endogenous tobacco myb factor controlling anthocyanin production.

### Cloning and expression of a putative tobacco homolog of *AtMYB90*

Alignment of the *AtMYB90* sequence against those contained in the tobacco transcription factor sequence database, TOBFAC, (<http://compsysbio.achs.virginia.edu/tobfac/>, [41]) identified a tobacco myb gene (gnl|tobfac|R2R3-MYB_141) with sequence similarity to the *AtMYB90* coding region. A PCR primer targeting the N-terminus of the predicted R2R3-MYB_141 coding region was designed and used to amplify and clone a cDNA for this putative tobacco *AtMYB90* homolog (PCR from start codon to a poly-A adaptor sequence, primers in Table 1)). The cloned tobacco Myb cDNA was sequenced and found to match that of a tobacco homolog (*NtAN2*) of the Petunia *AN2* myb gene recently added to the NCBI Genbank (FJ472647). In the spirit of standardized nomenclature we will refer to our tobacco myb homolog as *NtAN2*.

**Table 1:** PCR primers.

Set ID.	Forward Primer (5′->3′)	Reverse Primer (5′->3′)	Product (bp)
1	GGTTTACCCGCCAATATATCC	GACGCGTCGACGTCTTCTCGATCGTGTCGATCAATAC	523
2	CGGGCCTCTTCGCTATTAC	GACGCGTCGACGTCTTCTCGATCGTGTCGATCAATAC	389
3	GATCTTGAGCCAATCAAAGAGGAGTGATGTAGAC	AGCCCGATGACAGCGAC	656
4	GTACCGAGCCGCAGGAAC	AGCCCGATGACAGCGAC	273
5	TGGCATGACGTGGGTTTC	CCCTCTGGTCTTCTGAGACTGTATC	630
6	GATTCCATTGCCCAGCTATC	CCCTCTGGTCTTCTGAGACTGTATC	281
7	CCAACCACGTCTTCAAAGCA	ATCAAGTTCAACAGTCTCTCCATCA	1142/896 1
7a	CCAACCACGTCTTCAAAGCA	ACAAGTCAGGTATTATAGTCCAAGC	952
8	ACATAATATCGCACTCAGTCTTTCATC	TGCGAACGTTTTTAATGTACTG	602
9	ACATAATATCGCACTCAGTCTTTCATC	CGAGTGGTGATTTTGTGCCGA	732
NtAN2 (PCR)	ATGAATATTTGTACTAATAAGTCGTCGTCAG	AAAGATTAAATCCTACGTCTGCCTCATAAG	549
NtAN2 (cDNA)	TACCAAGACCATGGATATTTGTACT	ACAGGATCCTATCAACTGAAAAGTG	683
AtMybQ1 (qPCR)	GACTGCTGAAGAAGATAGTCTCTTG	GCCCAGCTCTCAAAGGAACTTGATG	104
NtMybQ1 (qPCR)	AGGCCACATATAAAGAGAGGAGACT	AATAAGTGACCATCTGTTGCCTAAC	107
icMGB (qPCR)	TCGCTAATGTGAGGACAGTGTA	ATCATCCATGTGCGTGGGACAGCAT	108
35S:: NtAN2	CACAATCCCACTATCCTTCG	AATAAGTGACCATCTGTTGCCTAAC	411
35S:: PG1Sh32	CACAATCCCACTATCCTTCG	TGTTTTTCTTTTTCATTTTAGACTT	511
NtAN2 In-Ex	AATGTAATTCTACTTATTGTAACAGGTACTTATC	CTTATGAAGCCTCAAAATGATGATCTAC	305

1 Two product sizes are indicated for Set 7 with the smaller number being associated with the PG-1Sh deletion construct.

A protein BLAST search using the *NtAN2* sequence identified *AtMyb113*, *75*, *90* and *114* genes (BLAST scores: 205, 194, 183, and 180) as the Arabidopsis proteins most closely related to *NtAN2*. All of these Arabidopsis Myb genes have been implicated in regulation of Anthrocyanin production and the next closest Arabidopsis gene in the search, transparent testa 2 (*TT2*, *AtMYB123*) is associated with proanthocyanin production in the seed coat. Consistent with a role as an activator of anthocyanin production in tobacco, qRTPCR analysis of *NtAN2* mRNA (primers listed in Table 1) detected *NtAN2* expression in flowers but none in leaf tissue (leaf Ct>35, at least 1000 fold less than flower mRNA levels [Ct∼23]). Further support for *NtAN2*′s role as a myb activator of anthocyanin production was provided by generation of transgenic *N. tabacum* (SR1) plants expressing a 35S::*NtAN2* transgene (the 35S::*NtAN2* construct substitutes the *NtAN2* coding region for that of *AtMYB90* in Fig. 1A). Several *NtAN2*-expressing R_0_ lines (12 of 71) displayed extensive ectopic purple pigmentation similar to patterns observed in tobacco lines expressing the 35S::*AtMYB90* transgene (e.g. Fig. 2A and 2B). Finally, transgenic tobacco plants expressing a double-stranded hairpin construct targeting the entire *NtAN2* coding region for RNAi (ihpNtAN2, a 35S::antisense-intron-sense hairpin within the pKO vector, [42]) was able to produce white flowers similar to those of PG-1 plants (2 of 12 lines showed a white flower phenotype, with the remaining lines displaying varying levels of pigment reduction, Fig. 2B and 3A). These findings are consistent with those reported by Pattanaik et al, at the ASPB Plant Biology Symposium, 2009 <http://abstracts.aspb.org/pb2009/public/P30/P30031.html>, and strongly suggest that *NtAN2* is a likely target for the interference with anthocyanin production seen in plants expressing the *PG-1* allele or PG-1Sh transgenes.

qRTPCR analysis of *NtAN2* gene expression in flowers from the set of representative PG-1Sh plants analyzed for PG-1Sh mRNA (Fig. 3A) did not indicate any correlation between flower *NtAN2* mRNA levels and anthocyanin pigmentation (R^2^ = 0.01). These results strongly suggest that PG-1Sh-associated interference in pigment production does not result from transgene-induced alterations in *NtAN2* transcription or from post transcriptional gene silencing of the *NtAN2* gene, leaving competitive protein-protein interaction as the most likely mechanism for the observed white flower phenotype.

**Figure 5:**
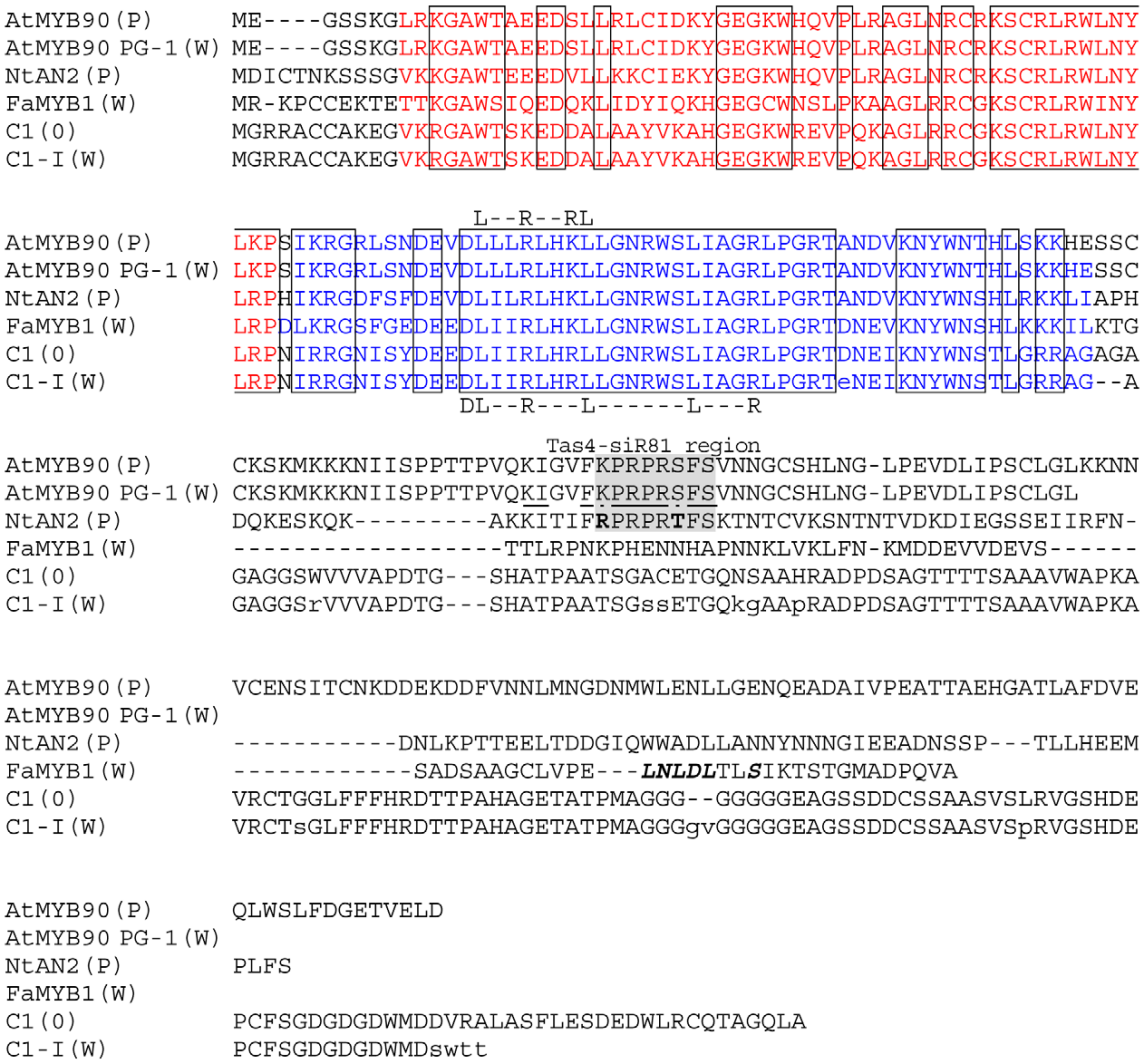
Protein sequence allignment (ProbCon, [44]) of R2R3 Myb proteins demonstrated to produce a reduction in anthocyanin phenotypes when expressed in transgenic tobacco. Ectopic over-expression of the indicated Myb genes produces extensive purple pigmentation (P), white flower phenotype (W) or no phenotypic change (0). The R2 repeat is indicated as Red text and R3 repeat as Blue. Amino acid sequences that align in all proteins are boxed, differences between C1 andC1-I are shown as lower case. Sequences associated with Myb-bHLH interaction (L--R--RL [49], DL--R---L------L---R [50]) are indicated above and below the aligned sequences. The amino acids encoded by the mRNA region of *AtMYB90* mRNA targeted by TAS4-siR81(-) are indicated by a grey box (**bold** indicates conservative amino acid differences between the *NtAN2* and *AtMYB90* sequences in that area). ***Bold-italic*** amino acids indicate the conserved C2 domain proposed to be important to *FaMYB1* repressor function [56]. Due to a native nonsense mutation the protein sequence of the *AtMYB114* allele in the Columbia ecotype is predicted to end 32 amino acids upstream from the PG-1 nonsense mutation (at the F residue just prior to the TAS4-siR81 (-) grey boxed region).

Alignment of the *NtAN2* cDNA with that of *AtMYB90* showed very little sequence similarity outside of that occurring within the 5′ repeats that are definitive of the R2R3 family of plant myb genes (Fig. 4). The only clear exception was a small region of sequence similarity just downstream from the R2R3 repeats (at ∼625 bp) which, interestingly, overlaps the area of the *AtMYB90* transcript targeted by an Arabidopsis trans-acting small interfering RNA [tasiRNA, specifically TAS4-siR81(−)] [43]. The tobacco sequence is not a perfect complement to the TAS4-siR81 (2 mismatches and a G::T pairing) and there is as yet no direct evidence suggesting that the observed sequence similarity reflects evolutionary conservation of a functional mRNA::siRNA interaction. In fact, alignment of the predicted amino acid sequences (Probcons, [44]) from the *NtAN2* and *AtMYB90* genes at the TAS4-siRN81 target site indicated only highly conservative amino acid substitutions (Arginine for Lysine and Threonine for Serine, Fig. 5) within a conserved nine amino acid segment. It is thus conceivable that the observed sequence similarity at the TAS4-siRN81 site is the result of an evolutionarily conserved protein function.

### The PG-1Sh version of *AtMYB90* also impacts anthocyanin production in transgenic 35S::*NtAN2* plants

**Figure 6:**
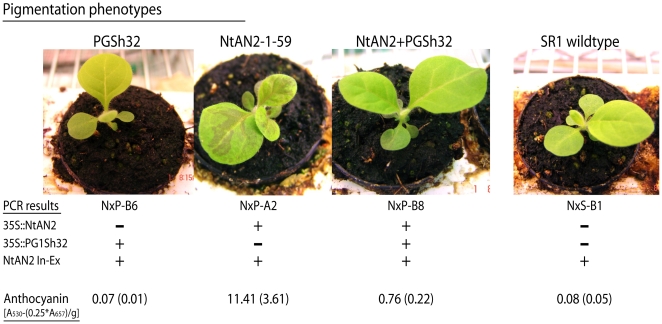
Representative anthocyanin pigmentation phenotypes for all possible transgene genotypes resulting from NtAN2-1-59 x PG-1Sh #32 crosses. The genotypes for each seedling (‘NxP’: NtAN2-1-59 x PG1Sh32, ‘NxS’: NtAN2-1-59 x SR1) are indicated below the photos as determined by PCR using primers that specifically target each transgene construct (35S::*NtAN2* or 35S::*PG-1Sh*). Primers targeting an intron-exon junction within the native tobacco *NtAN2* gene (NtAN2 In-Ex, Table 1) were used as a positive PCR control. Relative anthocyanin levels, determined using leaf tissues from each genotype, are listed below the PCR results (standard error for each measurement [n = 3 to 10] is shown in parentheses).

To confirm functional *in vivo* interaction between the PG1 and *NtAN2* gene products, PG-1Sh #32 transgenic plants were crossed with a 35S::*NtAN2* transgenic line (NtAN2-1-59) that displays enhanced anthocyanin production (Fig. 2A and 2B). The phenotypes (anthocyanin pigmentation) and genotypes (determined by gene-specific PCR, Table 1) of resulting F1 seedlings were compared (Fig. 6). As expected, plants containing only the 35S::*NtAN2* transgene displayed enhanced anthocyanin production within their leaves (Fig. 6). Seedlings containing both the 35S::*NtAN2* and *PG-1Sh* transgenes showed dramatically reduced anthocyanin production in leaves, in most cases appearing phenotypically identical to leaves from wildtype SR1 seedlings or plants containing only the *PG-1Sh* construct (Fig. 6). These data confirm the ability of the *PG1* gene product to interfere with *NtAN2* function in tissues other than flower petals, and indicate that the observed interference is independent of the promoter associated with *NtAN2* expression (the native *NtAN2* promoter drives expression in tobacco flower petals, while the virally derived CaMV-35S promoter controls *NtAN2* expression in NtAN2-1-59 transgenic leaves).

## Discussion

A single-base nonsense mutation within the coding region of an active Arabidopsis *AtMYB90* transgene (the *PG-1* allele) was found to convert the R2R3-myb gene from a transcriptional activator of plant-wide anthocyanin biosynthesis to a dominant-negative allele that was able to interfere with normal tobacco pigment production within flower petals. Confirmation that the *PG-1* gene product is responsible for the observed white-flower phenotype was provided by expression in transgenic tobacco of a truncated *AtMYB90* gene (*PG-1Sh*) engineered to produce the same shortened myb protein as that predicted for the mutant *PG-1* allele. The *PG-1Sh* transgenic lines displayed a range of flower pigmentation phenotypes, including white flowers similar to those seen with *PG-1* plants. Furthermore, anthocyanin content in representative *PG-1Sh* flowers was found to be inversely proportional to *PG-1Sh* transgene expression levels (Fig. 3A & 3B), supporting a negative function for the *PG-1Sh* gene product.

Based upon the highly pigmented phenotype of the Myb-27 tobacco line, the AtMYB90 protein is able to interact with those native tobacco transcription factors and promoters required to activate transcription of anthocyanin biosynthetic genes. This ability of an anthocyanin-associated myb factor to function in a non-native plant system is not unique, as similar pigmented phenotypes have been seen with ectopic over-expressed Myb transgenes in several heterologous plant species (e.g.: Maize *C1* expressed in tobacco; [33], Apple *MdMYB1* expressed in Arabidopsis [25]; Daisy *GMYB10* expressed in tobacco [23]; Arabidopsis *AtMYB75* expressed in petunia [26], tobacco [35] or tomato [31]; Sweet potato *IbMYB1* expressed in Arabidopsis [29]; Grape *VvMYB5a* expressed in tobacco [45]; and *Medicago truncatula LAP1* in legumes and tobacco [46]). The predicted PG-1 and PG-1Sh protein is a shortened version of the *AtMYB90* gene product, retaining the highly conserved R2R3 domains but lacking 78 amino acids at the C-terminus (Fig. 5).

Based on our results, the truncated PG-1 protein has lost the ability to induce pigment production but retained sufficient function to allow it to interfere with the tobacco anthocyanin regulatory system active in flower petals. The observed interference in flower anthocyanin biosynthesis does not appear to be the result of altered transcription or message stability (e.g. RNAi) of the presumed functional tobacco myb homolog (*NtAN2*) since steady-state *NtAN2* mRNA levels show no correlative relationship with *PG-1Sh* mRNA content or anthocyanin levels in transgenic flowers displaying a wide range of pigmentation (Fig. 3A).

A literature search identified two other examples of myb-based genes that effectively eliminate flower pigment production when over-expressed in tobacco, the *C1-I* allele from maize [37] and a wild-type strawberry myb gene (*FaMYB1* [38]). It was proposed that *FaMYB1* may act directly as a transcriptional repressor [38], while the mutant transcriptional activator, C1-I, was assumed to act as a competitor to a native tobacco Myb protein, replacing the native protein within specific transcription initiation complexes [37], [38], [47]. The high ratios of *PG-1Sh* to *NtAN2* expression seen in the least pigmented *PG-1Sh* transgenic flowers (∼40-fold PG-1Sh mRNA excess in the mostly white flower line #42 or ∼120-fold excess in the white-flower line #32], Fig. 3A), support a model that proposes competition between the ‘inactive’ PG-1 and ‘active’ NtAN2 proteins for a common site within anthocyanin-associated transcription complexes. A similar competitive inhibition of transcription complexes may explain the loss of pigmentation associated with over-expression of *AtMYB60* in lettuce [48]. The ability of an active PG-1Sh gene (PG-1Sh #32) to dramatically reduce anthocyanin production when crossed into the purple 35S::*NtAN2* transgenic line, NtAN2-1-59 (Fig. 6), further supports a model of protein competition since the observed interference occurs in non-flower tissues and affects *NtAN2* activity controlled by a promoter unrelated to that which regulates expression of the native *NtAN2* gene in flower petals.

Alignment of predicted C1, C1-I, FaMYB1, AtMYB90/PG-1 and NtAN2 protein sequences indicates that sequence similarity is primarily limited to the highly conserved R2R3 DNA-binding domains common to this family of plant myb genes (Fig. 5). All of the aligned anthocyanin-associated myb proteins do, however, share sequence motifs (Fig. 5) linked to myb-bHLH binding (L--R--RL [49], DL--R---L------L---R [50]). The presence of the conserved bHLH binding motif is consistent with possible competition between the dominant-negative PG1 gene product and NtAN2 protein for association with one or more tobacco bHLH proteins. Just downstream from the R2R3 domains there is a noticeable short segment of protein similarity between the *AtMYB90* and *NtAN2* sequences, KI--F[K/R]PRP[R/T]FS. This sequence overlaps with an active tasiRNA target site identified in the *AtMYB90* mRNA (TAS4-siR81−, [43]) and it is not clear whether the common amino acids represent a conserved protein domain or reflect a possible homologous tobacco tasiRNA target within the *NtAN2* message. Our current results do not directly support any interaction between the *PG*-1 and *NtAN2* genes at the level of mRNA regulation.

The simplest model for a competitive interaction between the PG-1 and NtAN2 myb proteins assumes that the 78 C-terminal amino acids missing in the *PG-1* product contain, or overlap with, a transcriptional regulatory domain required for gene activation. Although sequences downstream from the conserved R2R3 domains are generally assumed to contain protein sequences responsible for transcription activation and/or repression, very few specific motifs or functional domains have been confirmed in plant myb proteins (e.g. [11], [51], [52], [53]). Support for this model of plant myb protein function comes from work in which fusion of a 12 amino acid EAR repressor motif to the 3′ end of the AtMYB75 protein transformed the transcriptional activator into a gene specific repressor [54]. A search for conserved protein motifs in the AtMYB90, C1, NtAN2 and FaMYB1 protein sequences (online MEME analysis, [55]) failed to identify any motifs outside those already identified by protein alignment, specifically the R2, R3 domains, and for *AtMYB90* and *NtAN2*, the TAS4 target region. Specifically, the short conserved ‘C2’ motif (LNL[D/E]L-[G/S] [38], [56]), which contains the core EAR motif (LXLXL, [57]), present in the proposed myb repressor, FaMYB1 was not identified in any of the other myb protein sequences examined.

The PG-1 allele is the result of a spontaneous single-base mutation within a *AtMYB90* transgene that acts as a dominant-negative ‘repressor’ of pigment production in tobacco flowers. The *AtMYB114* gene present in the Arabidopsis Columbia ecotype (*AtMYB114* is one of three Arabidopsis genes with very high sequence similarity to the *AtMYB90* gene) contains a premature stop codon located 31 amino acids upstream from the PG1 mutation, and over-production of the AtMYB114 (Col) truncated myb protein was recently shown to negatively impact anthocyanin production in Arabidopsis [13]. Similar dominant-negative mutations that produce truncated Myb proteins have been identified as naturally occurring alleles of the maize C1 gene [58], [59]. Both gene systems demonstrate a potential evolutionary mechanism that can convert myb transcriptional activators into repressors. In the case of PG-1, repression of tobacco anthocyanin production appears to be the result of competitive inhibition of one or more tobacco myb proteins. This mechanism is different from that proposed for plant myb proteins that contain a functional repressor domain such as the conserved C2 domain [56] implicated in the regulatory function of *AtMYB4* [60] and *FaMYB1* [38], and should be considered as a possibility when plant myb genes are over-expressed to test their function *in vivo* [48]. The authors are unaware of any documented examples of native plant gene regulatory systems that use competitive inhibition by an ‘inactive’ R2R3 myb protein to down-regulate gene expression. It is, however, important that the potential for such regulatory mechanism be kept in mind when dissecting plant gene control pathways that make use of myb genes.

## Materials and Methods

### Gene constructs and stable plant transformation

Plasmids were prepared using standard cloning techniques [61] and appropriate DNA segments sequenced to confirm final constructs. When possible, different promoter, terminator, reporter and selectable marker cassettes were used within constructs to reduce the potential for recombination within plasmids. The 35S::*AtMYB90* constructs (T-DNA depicted in Fig. 1A) used the pPZP200 vector [62] modified to contain a glufosinate-resistance plant selectable marker near the T-DNA right border. The plant resistance construct consists of the bar gene coding region (552 bp) encoding phosphinothricin acetyl transferase (Accession number: AX235900), regulated by the peanut chlorotic streak virus promoter (240 to +1 bp) [63] and CaMV 35S transcript termination signal.

Transformation of tobacco (*N. tabacum* cv SR1) was accomplished using the *Agrobacterium tumefaciens* line EHA105 [64]. Plasmid constructs were electroporated into EHA105 as previously described [65] and transformation of tobacco carried out by the conventional leaf disc method [66], [67]. Regenerated transgenic shoots were rooted on MS-agar medium [68] containing B5 vitamins [69] and 500 µg/ml Claforan (sodium cefotaxime, Hoechst).

Callus was produced *de novo* from Myb-27 leaf tissue by placing surface sterilized material on MS-agar media supplemented with plant hormones (MS Salt; B5 Vitamins; Sucrose 2% [w/v]; indol-3-acetic acid (0.5 mg/mL); benzlaminopurine (0.5 mg/mL). After 2–3 weeks shoot production was induced by transfer of actively growing purple callus to the same media lacking indol-3-acetic acid. Shoots that displayed altered anthocyanin pigmentation levels or patterns were excised above the callus and moved to the same media lacking hormones for root induction and eventually transferred to soil.

### PCR and quantitative RT-PCR

Routine PCR used MJ Research PTC-100 thermocyclers (95°C-8 Min, 30 cycles-[94C-45 Sec, 56°C-30 Sec, 72°C-60 Sec], 74°C-5 Min) and reagents from Applied Biosystems®. Primer sets and product sizes are listed in Table 1.

Quantitative reverse transcriptase PCR (qRT-PCR, primers listed in Table 1) was performed using a LightCycler® 480 System and SYBR green kits (LightCycler® DNA Master SYBR Green I) from Roche Applied Science according to protocols provided by the manufacturer (2-step; 60°–72°, read once per second, ramp at 4.4°C/s up & 2.2°C/s down). Total RNA was prepared using either Ambion mirVana™ RNA isolation kits and suggested protocols or using Tri-Reagent® reagent from Ambion®. To control for potential variability in the biochemical processes that precede qRTPCR reactions, total RNA samples (5 µg each) were spiked with a synthetic control internal control (IC) mRNA (250 pg/reaction) produced in vitro using T7 RNA polymerase (using Ambion® MEGAscript® and MEGAclear™ kits) acting on a PCR product template (IC2r, Genebank Accession # GQ215228). Spiked samples were treated with RNAse-free DNAase (TURBO® DNase, from Ambion®) and cleaned post reaction as per manufacturer's instructions. Reverse transcription was performed using RETROscript® from Ambion® (following the manufacturer's protocols). Relative RNA values were calculated using formulas for ΔΔCt, the Pfaffl method [70], and according to Norgard, et al [71], applied to qRT-PCR data from total RNA samples (triplicate technical assays and the indicated number of biological replicates).

### Spectrophotometric anthocyanin assay

Anthocyanin levels were determined by extraction of soluble anthocyanins as described by Martin et al [72], and spectrophotometic measurement at 530 nm and 657 nm. The formula used for relative anthocyanin content is: A_530_-(0.25xA_657_)/g tissue extracted.
